# Health-Related Quality of Life Profiles among Patients with Different Road Traffic Injuries in an Urban Setting of Vietnam

**DOI:** 10.3390/ijerph16081462

**Published:** 2019-04-24

**Authors:** Hai Minh Vu, Anh Kim Dang, Tung Thanh Tran, Giang Thu Vu, Nu Thi Truong, Cuong Tat Nguyen, Anh Van Doan, Kiet Tuan Huy Pham, Tung Hoang Tran, Bach Xuan Tran, Carl A. Latkin, Cyrus S.H. Ho, Roger C.M. Ho

**Affiliations:** 1 Department of Trauma, Thai Binh University of Medicine and Pharmacy, Thai Binh 410000, Vietnam; vuminhhai777@gmail.com (H.M.V.); anhdv.ytb@gmail.com (A.V.D.); 2 Institute for Global Health Innovations, Duy Tan University, Da Nang 550000, Vietnam; cuong.ighi@gmail.com; 3 Center of Excellence in Evidence-based Medicine, Nguyen Tat Thanh University, Ho Chi Minh City 700000, Vietnam; tung.coentt@gmail.com (T.T.T.); giang.coentt@gmail.com (G.T.V.); 4 Center of Excellence in Behavioral Medicine, Nguyen Tat Thanh University, Ho Chi Minh City 700000, Vietnam; nu.coentt@gmail.com (N.T.T.); hocmroger@yahoo.com.sg (R.C.M.H.); 5 Institute for Preventive Medicine and Public Health, Hanoi Medical University, Hanoi 100000 Vietnam; phamhuytuankiet_vkt@fpt.vn (K.T.H.P.); bach.ipmph@gmail.com (B.X.T.); 6 Institute of Orthopaedic and Trauma Surgery, Vietnam—Germany Hospital, Hanoi 100000, Vietnam; tranhoangtung.vd@gmail.com; 7 Bloomberg School of Public Health, Johns Hopkins University, Baltimore, MD 21205, USA; carl.latkin@jhu.edu; 8 Department of Psychological Medicine, National University Hospital, Singapore 119074, Singapore; cyrushosh@gmail.com; 9 Department of Psychological Medicine, Yong Loo Lin School of Medicine, National University of Singapore, Singapore 119228, Singapore; 10 Biomedical Global Institute of Healthcare Research & Technology (BIGHEART), National University of Singapore, Singapore 119228, Singapore

**Keywords:** road traffic injuries, quality of life, Vietnam

## Abstract

Road traffic injuries (RTIs) cause a substantial disease burden in Vietnam. Evaluating health-related quality of life (HRQOL) among patients having a diversity of RTIs informs an integral part of treatment effectiveness. This study aims to examine HRQOL of patients suffering different RTIs in Vietnam’s urban areas. A cross-sectional study was conducted on 408 patients from October to December 2018 in six hospitals in Thai Binh. The EuroQol-5 dimensions-5 levels (EQ-5D-5L) and visual analog scale (VAS) were used to assess the HRQOL of patients. Multivariable Tobit regression was applied to measure the difference of HRQOL among different kinds of injuries. The mean EQ-5D-5L and VAS score was 0.40–0.66, respectively. Mean EQ-5D-5L index was lowest in patients with oral and facial injuries (0.22) and fracture injuries (0.23), while patients having hand injuries had the highest EQ-5D-5L index (0.54). EQ-5D-5L index had a negative association with oral, facial, and fracture injuries. Meanwhile, patients with brain, fracture, and multiple injuries tended to have lower VAS score. Poor HRQOL among patients injured in road traffic were observed. Pain management, early rehabilitation, and mental health counseling services should be considered during treatment time, especially among those having the brain, oral and facial trauma, fracture, and multiple injuries.

## 1. Introduction

Road traffic injuries (RTIs) have raised health concerns globally due to their substantial health and economic burden. RTIs rank eighth among the leading causes of death for all age groups [1], stand first in the causes of death for children and young adults who were 5–29 years old [2], and is forecasted to become the seventh leading cause of mortality worldwide [3]. In 2016, RTIs were responsible for approximately 1.35 million deaths and up to 50 million injured individuals [2]. It is estimated that the economic burden placed by RTIs is US $518 billion globally, accounting for 3% of most countries’ gross domestic product and is relatively high among low and middle-income countries [1,3]. People suffering from RTIs are more likely to have disabilities with long-term consequences rather than premature death [4]. This can be explained by the reduction of road fatalities and the increase of surviving likelihood after serious injuries [5].

The repercussions of RTIs are often acknowledged in terms of either physical injuries or psychological aspects which are based on the suddenness and violence as the nature of RTIs [6]. Health-related quality of life (HRQOL) should be indicated as a patient-reported outcome after suffering from RTIs. HRQOL has been used as a perceived health status which measures the effects of health problems and subsequent treatments on the physical well-being, psychological state, and social relationships [7]. The sequelae resulting from RTIs may decrease individual’s functional capability as well as work capacity [8], and patients may experience some psychological disorders such as posttraumatic stress disorder (PTSD), depression, and driving phobias [6,9]. Therefore, HRQOL can be utilized as an indicator for the recovery process after traumas.

In a previous study, people who suffered from road injuries reported that their perceived HRQOL was low (the mean EQ-5D utility score was approximately 0) and most of them had problems with mobility as well as performing usual activities [10]. Another study conducted among patients who underwent road traffic crashes also revealed that pain severity and interference with daily life were related to significantly lower HRQOL [11]. Previous studies show that socioeconomic characteristics, including older age, female gender, and occupation, were associated with poorer quality of life outcomes among people having RTIs [12,13]. Clinical characteristics were also considered as main factors, for example, higher initial pain, a greater number of symptoms, self-perceived, threating life, and participation in rehabilitation programs [14,15,16].

In Vietnam, the number of traffic accidents in urban areas is particularly high because of the heavy density of traffic, poor transport infrastructure, and inadequate physical road safety measures [17,18]. According to the report of the General Statistics Office of Vietnam, there were 18,232 traffic accidents, causing 8125 deaths in 2018, and approximately 50 traffic accidents occur daily, resulting in 22 deaths [19]. Currently, there is few literature with emphasis on how each type of trauma (soft tissue, hand, traumatic brain, oral and facial, spinal cord, chest, fractures, and multiple injuries) is associated with the quality of life of patients after suffering from RTIs. Hence, the current study aims to examine the quality of life and identify impacted factors among patients having traumas caused by RTIs in urban areas of Vietnam.

## 2. Materials and Methods

### 2.1. Study Setting and Sampling Method

A cross-sectional study was conducted from October–December 2018 in Thai Binh, Vietnam. The study settings included one provincial hospital (Trauma-Orthopedic/Burn department at Thai Binh Province Hospital) and five district hospitals (General Surgery Department at Kien Xuong, Hung Ha, Dong Hung, Quynh Phu, Thai Thuy district hospitals). Eligible participants were identified via the following criteria: (1) Aged 18 years old or above; (2) hospitalized due to suffering from traffic accidents; (3) received treatments at mentioned hospitals; (4) had the ability to communicate with the data collectors. Participants who suffered from severe injuries and were not able to answer the questionnaire were excluded from the study.

We applied the convenience sampling approach to recruit participants for the study. Participants were asked to be involve in the study when they attended the abovementioned hospitals for treatment services. Data from participants were obtained via face-to-face interviews. The interviews were carried out in a private counseling room and participants answered the questionnaire after being informed about the purposes, benefits, and drawbacks of the study. All the participants in the study were introduced to and signed the written informed consents. We also utilized the medical records to obtain information regarding the clinical characteristics of the participants. Interviewers were health professionals, including medical doctors and nurses from hospitals. They were trained by professionals in conducting interviews to ensure the quality of data. In order to secure the text and logical issue of each question, the questionnaire was piloted in 50 patients and only a few questions were modified because of unclear meaning. A total of 408 patients agreed to participate in the study.

### 2.2. Measurements and Instruments

#### 2.2.1. Socioeconomic Characteristics

The participants self-reported their information regarding age, gender, level of education, marital status, occupation, as well as monthly income. In addition, we asked the participants whether they had health insurance.

#### 2.2.2. Injury-Related Characteristics

The participants self-reported whether they had self-accidents and used protective equipment when having accidents. Data imported from medical reports were types of injuries, which were classified into eight categories, including traumatic brain, oral and facial, chest, spinal cord, soft tissue, hand, limb fractures, and multiple injuries. Oral and facial injuries included soft tissue injuries, nasal injuries, and fractures. Soft tissue injuries ranged from minor abrasions and bruising of skin to major traumas such as disruption of tendons, ligaments, and muscles. Hand injury contained traumas related to the wrist, hand, and finger (all structures of soft tissue, nerves, blood vessels, bones, and joints). Hand injury was classified as a specialized trauma because hand surgeons require a special technique such as microsurgical reattachment or microsurgical reconstruction of soft tissues and bone, nerve reconstruction, and surgery to improve the function of upper limbs [20]. Limb fractures were the fracture of the bones of arms, forearms, femurs, kneecaps, shins, and feet.

#### 2.2.3. Health-Related Quality of Life (HRQOL)

In this study, HRQOL was examined using the EuroQol-5 dimensions-5 levels (EQ-5D-5L) [21]. Five subgroups were examined in this scale including mobility, self-care, usual activities, pain/discomfort, and anxiety/depression. Each dimension was rated by a Likert scale with five response levels from no problem to extreme problems. EQ-5D-5L then was transformed into an index score using a Vietnamese cross-walk value set [1]. Moreover, in order to assesss the self-rated quality of life of participants within the interview day, we utilized a visual analog scale (VAS). This tool was scored from 0 (the worst health condition) to 100 (the best health condition).

### 2.3. Statistical Analysis

Data were analyzed using STATA 15.0 (Stata Corp. LP, College Station, TX, USA). We used descriptive statistics to demonstrate variables including frequency, percentage and mean, and standard deviation (SD). Multivariate Tobit regression models were used to identify associated factors with HRQOL of respondents. Independent variables in the regression model included socioeconomic characteristics, self-accident, use of protective equipment, and types of trauma. Outcome variables were EQ-5D-5L index (value of the index ranged from −1–1) and VAS score (score ranged from 0–100). A *p*-value <0.05 was considered statistically significant.

### 2.4. Ethics Approval

The study protocol was assessed and approved by the Institutional Review Board of Thai Binh University of Medicine and Pharmacy. The participants’ information was only used for research and kept confidential (ethics approval code: 7642/HĐĐĐ).

## 3. Results

There was a total of 408 respondents in the study. Most of them had high school or higher education (53%), lived with a spouse/partner (72.3%), were blue-collar workers (54.7%), and had health insurance (89.5%). The mean age was 45.5 (Standard Deviation (SD) = 17.0). The average monthly income of respondents was US $381 (SD = 230.8) (Table 1).

Table 2 shows the traffic injuries characteristics of participants. Out of all the participants, 45.6% caused accidents by themselves, and 83.5% used protective equipment during the time accidents occurred. The most common types of injuries were fracture (35.1%), soft tissue (27.7%), and traumatic brain (18.6%). The mean EQ-5D-5L index score was 0.40, and the mean VAS score was 0.66. EQ-5D-5L index was lowest in patients with oral and facial injuries (0.22) and fractured injuries (0.23). Meanwhile, patients with hand injuries had the highest EQ-5D-5L index (0.54). The VAS score of patients with multiple injuries, spinal cord and traumatic brain injuries were 0.56–0.58, respectively. Patients with soft tissue and hand injuries were reported to have the highest VAS score (0.69).

**Figure 1:**
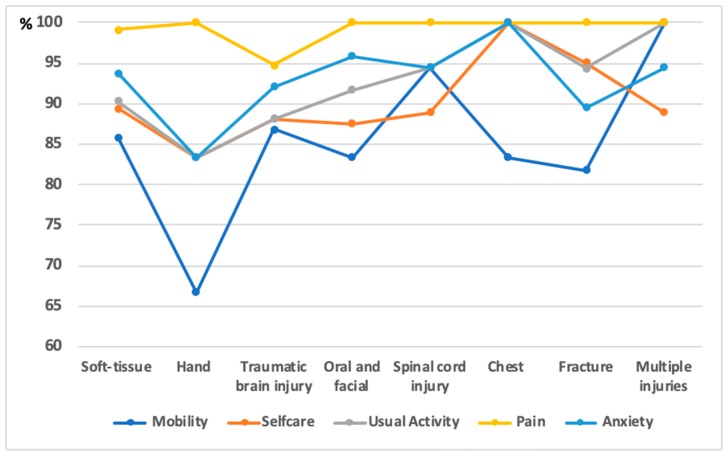
Health problems among the patients with different types of traffic injury.

Figure 1 illustrates the health problems among the patients with different types of injuries. The majority of the patients felt painful. The percentage of patients with injured hand were met with difficulty in mobility, self-care, and performing usual activity, and experienced anxiety was lowest compared to other types of injuries. Meanwhile, 100% of patients with multiple injuries had problems with mobility and usual activity. Patients experiencing traumatic brain injury and spinal cord injury had a high percentage of having all health problems. All of the patients with chest injuries had a problem with self-care and experienced anxiety/depression.

Table 3 indicates factors associated with HRQOL of respondents. The results show that patients with self-accident were more likely to have a higher score of EQ-5D-5L index and VAS. Participants who used protective equipment when accidents occurred tended to have higher VAS score. Patients with fracture injuries were associated with having lower either EQ-5D-5L index or VAS score. The EQ-5D-5L index score of patients had a negative association with oral and facial injuries. Meanwhile, patients with traumatic brain and patients multiple injuries were more likely to have lower VAS score. We also found that patients with hand injuries were more likely to have higher EQ-5D-5L index score.

## 4. Discussion

This study found low quality of life scores among patients who experienced RTIs, especially those having oral and facial trauma, as well as fracture. The majority of patients suffered from all health problems including physical and mental health issues. Factors negatively affected with HRQOL score were having traumatic brain injuries, oral and facial trauma, fracture, and multiple injuries. This is one of the first study contributing critical insights into HRQOL of people having RTIs in Vietnam, offering evidence for adopting changes in health strategies and further health interventions.

The mean EQ-5D-5L index score in our study was 0.4, which was lower than the utility scores of Vietnamese populations measured by EQ-5D-5L [21]. Compared to a previous study conducted among patients having motor vehicle crash in New South Wales, HRQOL score in our study was lower in both EQ-5D-5L and VAS scales [12]. The differences can be explained by the fact that participants in the previous study were successfully claimed the payment for care and rehabilitation, as well as compensations for the loss of earnings [12], which positively associated with physical and mental well-being [22]. The previous study also revealed that patients with mild injuries had lower levels of quality of life until three years after the trauma, while lower physical abilities, cognitive functions, and self-esteem were the factors having negative effects to the quality of life of patients [23]. Our reported HRQOL score was also lower in comparison with results of a study examining quality of life among patients suffering from occupational injuries [24]. The majority of patients in such studies had undergone traumatic limb injuries which are less dangerous and less severe than other injuries, such as traumatic brain and oral-maxillofacial injuries [25,26]. Moreover, in our study, participants with hand injuries also reported the highest quality of life score while those suffering from oral and facial injuries and fractures had the lowest score.

In this study, it was recorded that a large proportion of participants had health problems in terms of both physical and nonphysical dimensions. Pain was a remarkable factor in this study that almost all participants had to face. This is consistent with previous studies which indicated that experiencing pain/discomfort was commonly long-lasting after an injury and major trauma [27,28,29]. Pain was considered as a reason for nonrecovery after injuries [30] and the presence of pain was the symptom of poor psychological well-being, it may also trigger a circle of events which lead to poor HRQOL and exacerbate other mental health issues [31]. Traumatic brain, spinal cord, and multiple injuries resulted in a large number of health issues, including mobility, self-care, performing usual activity, and anxiety. It remains a fact that suffering from the abovementioned injuries would be a life-changing experience for many patients because they would have to deal with disabilities or long-term consequences regarding cognitive or “thinking” tasks associated with memory [26,32], and loss of movement or sensation [33,34,35].

In this study, we also found that the lack of protective equipment when the accident occurred and traumatic brain injuries were negatively associated with HRQOL score. In Vietnam, motorcycles are the principal means of transportation, and motorcycle users are highly vulnerable to RTIs [32]. According to the World Health Organization, not wearing protective equipment such as helmets is one of the main cause of traumatic brain injury which primarily contributes to fatal injuries [36,37]. Researchers have reiterated that the constraints of physical, psychological, and social problems after traumatic brain injuries present massive challenges to the rehabilitation and the reintegration into society of patients [38,39]. Physical health problems directly affecting the brain such as epilepsy, postconcussion symptoms, or mental problems caused by low life-satisfaction levels may have a great impact on HRQOL of patients [23,40]. Oral and facial trauma, fracture and multiple injuries were also related to lower score of HRQOL among RTI patients.

Several implications could be drawn from this study. In term of clinical treatment, the injury pain outcome should be noticed and treated effectively during the recovery process. Pain management for RTI patients is crucial as it may foster psychological well-being, reduce morbidity, and improve long-term outcomes. Moreover, the fact that the participants exhibited low levels of quality of life and had many health problems suggests the importance of implementing healthcare services that assist patients in early rehabilitation and provide necessary mental health counseling services. This may help patients elevate their functional capacity and ability to reintegrate into society. Patients experiencing certain types of trauma such as traumatic brain injury, oral and facial trauma, fracture, and multiple injuries should be promptly provided special support and attention from caregivers due to the severity and higher risk of having lower HRQOL scores. Finally, findings from our study can be used as critical evidence for decision makers to monitor the adjustment of health policies and allocate financial resources for healthcare among people having RTIs. Likewise, these data can be considered as a reference for health economists to evaluate the difference of HRQOL amongst specific populations, which may help to calculate quality-adjusted life years in the economic assessment in various Vietnamese settings.

Nevertheless, several limitations should be acknowledged. First, it is difficult to draw causal conclusions due to the characteristics of the cross-sectional study design. Second, recall bias and social desirability may have led to under- or overestimation of some responses. Finally, the convenience sampling technique may have limited the ability to generalize the results of the study for the whole population of Vietnam. Further studies which determine the preference weights for EQ-5D-5L utility index are necessary to examine the HRQOL of the population more accurately. Moreover, studies using different study design, as well as various data collectors, should be implemented to suppress the social desirability, and findings from these studies can be used as evidence to compare with our study results.

## 5. Conclusions

In summary, we observed a low level of HRQOL among patients suffering from RTIs. Pain management, early rehabilitation, and mental health counseling services should be carefully considered during treatment time, especially among those having the traumatic brain, oral and facial trauma, fracture, and multiple injuries. The future budget on healthcare for people with RTIs should be given more attention and be efficiently allocated by decision makers.

## Figures and Tables

**Table 1:** Socioeconomic characteristics of respondents.

Characteristics	Total	
	*n*	%
	408	100
**Education**		
Under high school	192	47.1
High school	139	34.1
Above high school	77	18.9
**Marital status**		
Single	113	27.7
Have spouse/partner	295	72.3
**Employment**		
Student	20	4.9
Blue collar	223	54.7
White collar	25	6.1
Freelancer	84	20.6
Others	56	13.7
**Health insurance**		
No	43	10.5
Yes	365	89.5
	**Mean**	**SD**
**Age**	45.5	17.0
**Monthly income (USD)**	381.5	230.8

**Table 2:** Traffic injury characteristics of the participants.

Characteristics	Total		EQ-5D-5L Index		VAS	
	*n*	%	Mean	SD	Mean	SD
Self-accident	186	45.6				
Used protective gear when the accident occurred	228	83.5				
Type of injuries						
Soft tissue	113	27.7	0.33	0.38	0.69	0.39
Hand	18	4.4	0.54	0.25	0.69	0.10
Traumatic brain injury	76	18.6	0.30	0.43	0.58	0.19
Oral and facial	24	5.9	0.22	0.40	0.61	0.18
Spinal cord injury	18	4.4	0.34	0.41	0.57	0.20
Chest	12	2.9	0.48	0.29	0.61	0.14
Fracture	143	35.1	0.23	0.38	0.66	0.36
Multiple injuries	18	4.4	0.35	0.32	0.56	0.17
Total			0.40	0.37	0.66	0.26

**Table 3:** The factors associated with HRQOL of respondents.

Variables	EQ-5D-5L Index		VAS	
	Coef. (95% CI) ^a^	Coef. (95% CI) ^b^	Coef. (95% CI) ^a^	Coef. (95% CI) ^b^
Self-accident	0.10 (0.03; 0.17) *	0.10 (0.03; 0.18) *	0.05 (0.01; 0.08) *	0.04 (0.01; 0.08) *
Used protective equipment when the accident occurred	0.06 (−0.06; 0.18)	0.08 (−0.04; 0.20)	0.05 (−0.00; 0.11)	0.06 (0.00; 0.11) *
Type of injuries				
Soft tissue	−0.05 (−0.13; 0.03)	−0.04 (−0.12; 0.04)	0.02 (−0.02; 0.05)	0.01 (−0.03; 0.05)
Hand	0.18 (0.01; 0.36) *	0.17 (−0.00; 0.34) *	0.05 (−0.04; 0.13)	0.04 (−0.04; 0.12)
Traumatic brain	−0.08 (−0.17; 0.01)	−0.08 (−0.18; 0.01)	−0.09 (−0.13; −0.04) *	−0.09 (−0.13; −0.04) *
Oral and facial	−0.16 (−0.31; −0.00) *	−0.15 (−0.31; 0.00) *	−0.05 (−0.12; 0.03)	−0.04 (−0.11; 0.03)
Spinal cord	−0.03 (−0.21; 0.15)	−0.02 (−0.20; 0.16)	−0.09 (−0.17; −0.00) *	−0.07 (−0.15; 0.02)
Chest	0.12 (−0.10; 0.33)	0.11 (−0.10; 0.33)	−0.04 (−0.14; 0.06)	−0.03 (−0.13; 0.07)
Fracture	−0.20 (−0.28; −0.13) *	−0.20 (−0.27; −0.13) *	−0.04 (−0.07; −0.00) *	−0.04 (−0.07; −0.00) *
Multiple injuries	−0.01 (−0.19; 0.16)	0.01 (−0.16; 0.19)	−0.09 (−0.17; −0.01) *	−0.09 (−0.18; −0.01) *

^a^ Crude coefficient; ^b^ Adjusted to age, employment, gender, living area, education, marital status, and monthly income. * *p* < 0.05.
